# Computational mechanobiological model combining epiphyseal, apophyseal, and appositional growth and inner bone remodeling of the juvenile femur

**DOI:** 10.1186/s12938-026-01598-3

**Published:** 2026-07-15

**Authors:** Andreas Lipphaus, Ralf-Bodo Tröbs, Matthias Klimek, Sascha Selkmann, Ulrich Witzel

**Affiliations:** 1 Biomedical Engineering Lab, Ruhr University Bochum, Bochum, Germany; 2 Clinic of Pediatric Surgery, St. Mary’s Hospital Witten, Witten, Germany; 3 Department of General-, Visceral- and Pediatric Surgery, St. Vinzenz Krankenhaus Paderborn, Paderborn, Germany; 4 Clinic for Trauma, Hand and Reconstructive Surgery, Institut für Muskuloskelettale Medizin, University Hospital Münster, Münster, Germany

**Keywords:** Biomechanics, Finite element, Bone growth, Endochondral ossification, Growth plate, Femoral neck isthmus, In silico modeling, Pediatric orthopedics

## Abstract

In silico models for simulating bone growth based on mechanical or non-mechanical epigenetic factors are widely used. In this study, a well-known mechanobiological model, which states that octahedral shear stress accelerates longitudinal bone growth and hydrostatic stress retards it, is applied to a finite element model of the femur of an 8-year-old boy. Proximal and distal epiphyseal plates as well as the growth plate of the greater trochanter, cartilaginous growth at the femoral isthmus, and appositional bone growth are included in the model. Furthermore, changes in the density of the cancellous bone in the metaphyses are modeled based on Wolff's law using compressive stresses as the mechanical stimulus. Muscle forces during a dynamic gait cycle were determined for nine discrete loading cases by optimizing to minimize bending stress. The highest stresses in the femoral shaft were determined as medial compressive stresses with a maximum of −33.2 MPa. Highest internal axial load in the shaft was 985 N during loading response. The simulated bone growth resulted in an increase in femur length of 23.7 mm and a decrease in femoral neck angle by −1.4°, anteversion angle by −2.4°, and lateral distal femur angle by −1.6° per year. Growth of the apophysis of the greater trochanter resulted in an unchanged articulo-trochanteric distance. The bone remodeling led to an increase in bone density, particularly in the medial proximal metaphysis. The consideration of different growth mechanisms allowed a comprehensive simulation of femoral growth with high agreement with anthropometric data. Possible applications are the simulation of the correction of deformities.

## Introduction

In children, longitudinal bone growth occurs at the cartilaginous epiphyseal plates by endochondral ossification [1]. These growth plates are located at the end of the long bones between the metaphysis and the epiphysis and can be divided into horizontal zones. Next to the epiphysis, the resting zone contains stem-like cells and secretes morphogens influencing the alignment of the proliferative chondrocytes and inhibiting their terminal differentiation [2]. The finite proliferative capacity of resting chondrocytes leads to growth deceleration and finally epiphyseal fusion by growth plate senescence as children age [3]. The resting zone is followed by the proliferation zone, where chondrocytes rapidly divide, form columns of flattened, disc-shaped chondrocytes, and synthesize new matrix [4]. Toward the end of the proliferative zone, chondrocytes enlarge and take a more spherical shape forming the hypertrophic zone [5]. Two main hypotheses of ossification at the end of the hypertrophic zone involve either the apoptosis of hypertrophic chondrocytes followed by invasion of osteoblast precursors by the vascular system or transdifferentiation of chondrocytes to osteoblasts [6]. Appositional growth increases the thickness or diameter of the bone. In this mechanically stimulated process, osteoblasts deposit new matrix on the periosteal and endosteal surfaces and differentiate into osteocytes [7].

Bone growth in children is orchestrated by various mechanical [8, 9] and non-mechanical factors like inflammation [10], growth [11] and sex hormones [12], and genetics [13]. Several studies investigated the rate of longitudinal growth in the growth plates as a function of static or dynamic loading. The Hueter–Volkmann law states that epiphyseal growth is increased by tensile stress and retarded by compressive stress acting at the growth plate [14]. While in some animal models only minor femoral length differences are reported due to different physiological dynamic loadings [15], a growth retardation of 52–63% is observed in paralyzed embryonic chicks due to a lack of mechanical stimulation [16]. In animal models, a linear relationship between sustained tensile and compressive stresses and acceleration and deceleration of growth was observed [17]. This effect is due to reduced numbers of both proliferative and hypertrophic chondrocytes and increases approximately linearly with time [18]. The effect of cyclic or intermittent loading is still not fully understood: while some studies found no significant bone length differences with different exercises in rats [19], others reported an increase in the length of radius and ulna in the dominant arm of professional tennis players [20]. Smith experimentally determined the principal stress directions in the epiphyses and concluded that the stress trajectories often are orthogonal to the direction of bone growth [21]. A second hypothesis is that due to the low stiffness of the cartilage under load, a deformity occurs and that growth proceeds in the direction of the distortion of the cartilage columns as long as this is too small to lead to tissue damage [22–24]. Pauwels proposed that deformities, especially after fractures, lead to asymmetric growth by uneven loading of the epiphyseal plate and thereby works to correct these deformities [25]. Carter and Wong proposed a mechanobiological model that epiphyseal growth is inhibited by dynamic hydrostatic compression and accelerated by octahedral shear stress. They defined the osteogenic index as the sum of maximum octahedral shear stress and minimum hydrostatic stress overall load cases and calculated the resulting growth as the product of the osteogenic index and the mechanical growth rate [26].

Endochondral growth primarily causes functional adaptations through angular adaptations till the fusion of the growth plates. In contrast, bone formation and resorption optimize bone density and volume, especially in childhood, but also in adults. Appositional growth occurring at the periosteum is influenced by local strains [27]. Higher loading is associated with greater periosteal growth [28]. Thereby, the bone's diameter increases, and not only the axial strength but in particular the strength of bone in bending and torsion is increased. Indeed, previous finite element simulations suggested that torsional load is the most important stimulus in forming the medullary cavity [29]. In adults, periosteal formation is seen in regions stressed with more than 20 MPa [30] or 1500 με [31]. Above a threshold of about 4000 με overload resorption occurs and bone density decreases. In growing bones exercise leads to a higher cortical area, while disuse results in decreased cortical area compared to normal developing bones [32]. However, due to the lower Young's modulus, higher deformations occur [33]. In addition, the threshold of bone formation is lower. A study in adolescent rats has reported significant and prolonged bone formation with strains of 850 με [34]. Overall, mechanically controlled growth patterns allow the bone to adapt to its mechanical needs. Thereby, bending stresses can be reduced to achieve lightweight design [30, 35, 36].

Based on finite element models, several computational approaches were established to simulate bone growth due to metabolic and mechanobiological signaling. Early models depicted either the biochemical or the mechanobiological component of growth. Heegard et al. used a pure mechanobiological model to simulate prenatal joint development [37], bone straightening [38], and epiphyseal growth of the distal femur [39]. Based on this, the development of bone deformities resulting from pathologies such as cerebral palsy [40], cam deformity [41], and developmental dysplasia of the hip [42–44] has been simulated. A similar approach was developed by Stokes et al. and uses tensile or compressive axial stresses to describe the longitudinal bone growth [45]. Alonso et al. presented a mechanobiological model simulating epiphyseal growth and remodeling after virtual implantation of staples for hemiepiphysiodesis [46]. In the latest models, reaction–diffusion equations of morphogens and mechanobiological approaches were combined to model the embryonic development of synovial joints and the femur [47–49]. Also, changes in bone density of the trabecular bone in the proximal femur of children have been modeled [50, 51], resulting in an increase in bone mineral density on the medial side of the femoral neck [52].

The proximal femur has three growth plates: the growth plate located between the metaphysis and the femoral head, the trochanteric growth plate, and the femoral neck isthmus [53]. Because chondral growth is faster than periosteal growth, the cartilage of the femoral neck isthmus is necessary to compensate for length growth and shape the femoral metaphysis. The growth of the trochanteric apophysis affects the statics of the hip joint as the hip resultant force is mainly defined by the body weight and the hip abductors attached to the greater trochanter. The force resultant at the greater trochanter is formed by forces through a muscle sling of the vastus lateralis with the opposing gluteus medius and minimus and the iliotibial band [54], reducing bending in both the femoral shaft and near the hip joint. Growth of the greater trochanter results in a reduction of the femoral neck–shaft angle [55], and subsequently, epiphysiodesis of the greater trochanter has been described as a treatment for infantile coxa vara [56]. The morphological changes during growth allow the bone to maintain physiological loading and a constant cartilage load [57].

Current in silico models either focus on very early phases of bone development or focus on a single growth mechanism. For transition into clinical practice, a more comprehensive model is needed. As the greater trochanter plays an important role in the biomechanics of the hip, it is of interest to include its growth in in silico models of bone development. Therefore, this study aims to develop a mechanobiological model of the growth of the juvenile femur including all growth plates, appositional growth of the femoral shaft, and inner remodeling. Stresses and internal axial loading are calculated during gait and muscle forces are optimized to reduce bending stresses. For validation, growth predicted by the in silico model is compared to several in vivo anthropometric measurements.

## Results

### Optimization of muscle forces

**Fig. 1:**
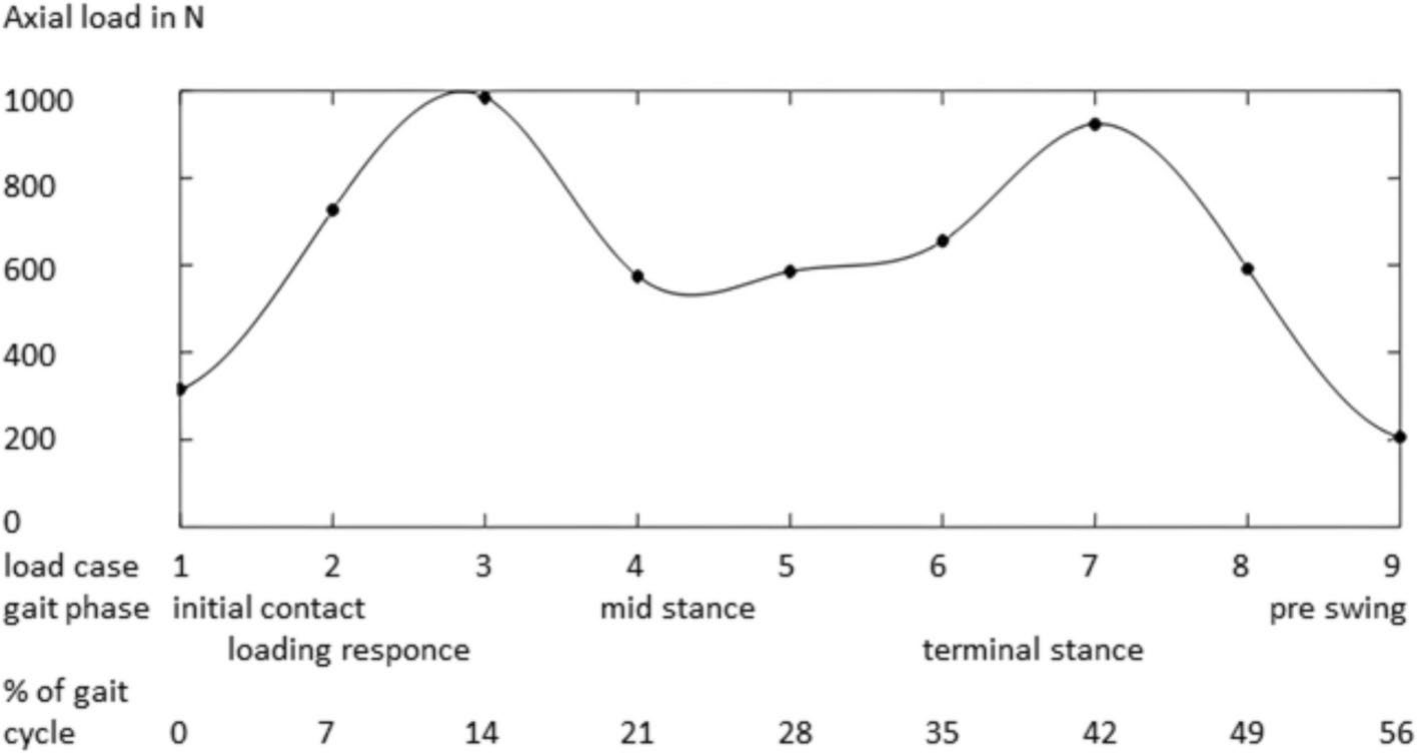
Course of the axial load for all nine load cases. The respective phases of the gait cycle are shown accordingly. Axial forces range between 200 and 985 N at midshaft with peaks at the end of loading response and terminal stance

Before the growth simulation was initiated, muscle forces were calculated using a bending minimization approach (Table 1). Two peaks in axial load at midshaft, the first during loading response and the second during terminal stance (Fig. 1) were associated with peaks in muscle forces of the gluteal muscles and the iliopsoas and iliacus muscles, respectively.

**Table 1:** Resultant muscle forces in Newton (N) for all nine load cases in an 8-year-old boy using a bending minimization approach. Muscle forces are rounded to full numbers. Forces below 1 N are considered as no muscle activation (-). Additionally, resulting axial loading is given for all load cases

Muscle/load case	1	2	3	4	5	6	7	8	9
Adductor brevis	4	–	–	–	–	–	–	–	32
Adductor longus	1	–	–	–	–	–	–	2	34
Adductor magnus	10	52	–	–	–	–	–	–	–
Biceps femoris	45	–	–	–	–	18	96	64	37
Gastrocnemius lateralis	11	–	–	–	–	55	125	37	–
Gastrocnemius medialis	9	–	–	–	–	123	372	32	–
Gemellus	-	2	6	5	3	9	9	–	12
Gluteus maximus	11	188	235	152	35	2	–	–	-
Gluteus medius	–	131	609	368	282	266	206	109	23
Gluteus minimus	–	10	64	27	27	59	60	57	12
Iliacus	–	–	–	–	–	137	880	524	244
Pectineus	–	–	–	–	–	–	–	–	5
Piriformis	–	14	62	17	8	29	67	12	29
Psoas	–	–	–	–	–	3	208	64	169
Quadratus femoris	11	4	2	–	–	–	–	–	–
Vastus intermedius	–	23	47	28	13	–	–	–	–
Vastus lateralis	–	100	191	94	16	–	–	–	–
Vastus medialis	–	45	86	43	8	–	–	–	–
Axial loading	316	727	985	575	586	656	924	592	206

**Fig. 2:**
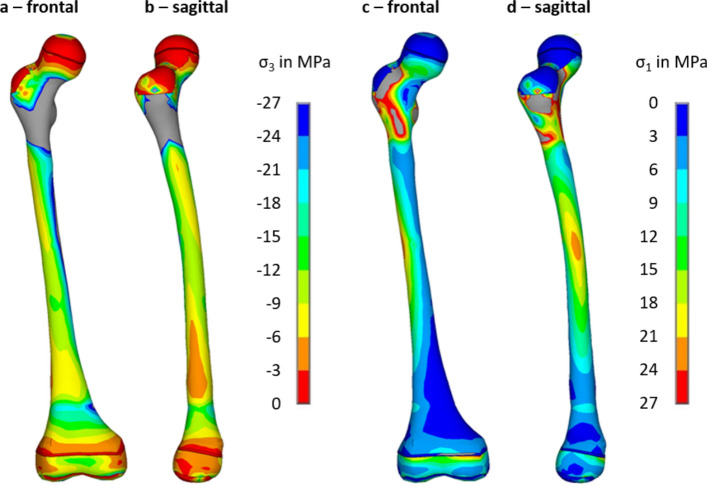
Views of superposition of stresses of all nine load cases illustrate loading of cortical bone: 3rd principal stresses σ_3_ in MPa in frontal (**a**) and sagittal (**b**) views highlighting compressive stresses and 1st principal stresses σ_1_ in MPa in frontal (**c**) and sagittal (**d**) views highlighting tensile stresses in cortical bone. The stresses are greatest at the proximal metaphysis. In the femoral shaft, 3rd principal stresses peak medial and 1st principal stresses lateral. Most parts of the cortical bone exhibit compressive stresses between −6 and −27 MPa

Finite element analysis of the physiological superposition of all nine load cases in the initial model of the femur of an 8-year-old shows that 3rd principal stresses are highest in the proximal femur on the medial side (Fig. 2a, b). In contrast, compression in the distal femur is mostly homogeneously distributed. In the shaft, compression is minimal on the anterior (−10.9 MPa) and lateral side (−11.7 MPa), whereas higher values are predicted on the posterior (−21.1 MPa) and medial (−33.2 MPa) side. 1st principal stresses are opposite to the compressive stresses (Fig. 2c, d). Values are generally lower with tensile stresses of 10 MPa anterior, 21.1 MPa lateral, 6.6 MPa posterior, and 5 MPa medial. Before optimization of muscle forces but including the iliotibial band and using physiological boundary conditions bending stresses are 24 MPa in the sagittal and 24.9 MPa in the frontal plane. After optimization, bending stresses in the frontal plane decrease by 78% while increase in the frontal plane by 9%. Loading results in axial forces between 200 and 985 N at midshaft with minimal shear loading. Axial loadings peak at the end of loading response and terminal stance. Hip and knee joint resultant is comparable to subject-specific musculoskeletal models in literature [58].

**Fig. 3:**
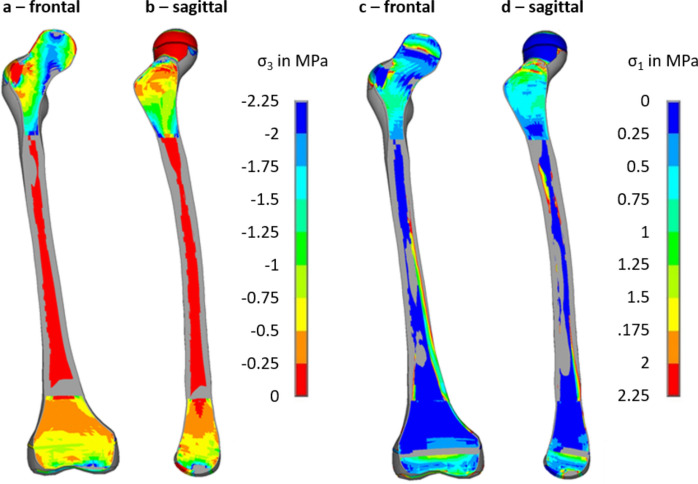
Sections of superposition of stresses of all nine load cases illustrate loading of trabecular bone: 3rd principal stresses σ_3_ in MPa in frontal (**a**) and sagittal (**b**) sections highlighting compressive stresses and 1st principal stresses σ_1_ in MPa in frontal (**c**) and sagittal (**d**) sections highlighting tensile stresses in cortical bone. At the proximal metaphysis, 3rd principal stresses peak medial and 1st principal stresses lateral. Most parts of the trabecular bone exhibit compressive stresses between −0.25 and −2.25 MPa

Cross sections show corresponding stresses in the trabecular bone (Fig. 3). In the frontal section, the highest compression is observed in the proximal femur on the medial side, while tension peaks at the lateral side. As in cortical bone, the trabecular bone of the distal femur is more uniformly stressed. Of note, the compression near the distal growth plate is slightly higher anterior and lateral. Other than that, in the sagittal section compressive and tensile stresses are mostly homogeneously distributed.

### Simulation of physiological growth

**Fig. 4:**
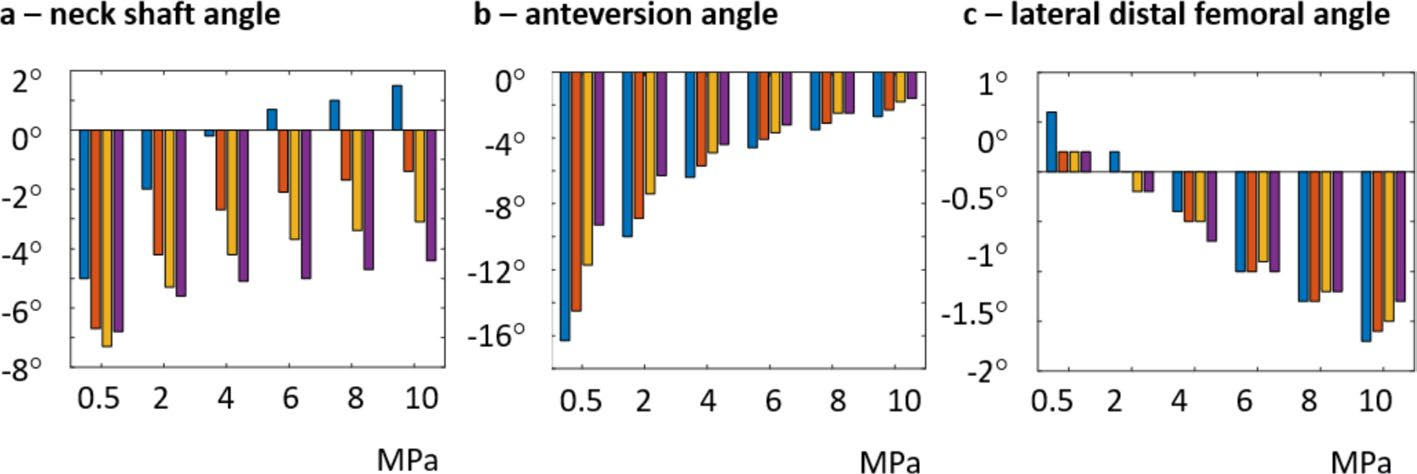
Plots of the absolute change in ° per year in NSA (**a**), anteversion angle (**b**) and LDFA (**c**) depending on the Young´s modulus and *b*/*a* ratio of 0.3 (blue), 0.5 (orange), 0.7 (yellow), and 1 (purple). As the b/a ratio increased, the reduction in the neck-shaft angle became more pronounced, whereas the decrease in the anteversion angle became smaller. Lower Young’s modulus values were associated with greater reductions in both angles

The predicted growth direction of the proximal femur is highly dependent on Young’s modulus of the growth plate. An increase in the b/a ratio was associated with a greater decrease in the neck–shaft angle, while the decrease in the anteversion angle was smaller at higher values (Fig. 4). Lower values of the Young’s modulus of the epiphyseal plates resulted in a greater reduction in both the anteversion angle and the neck–shaft angle. The LDFA showed with a *b*/*a* ratio of 0.3 a decrease for a low Young’s modulus and an increase for higher stiffness. For higher *b*/*a* ratios, higher stiffness resulted in a more pronounced decrease of the LDFA. An appositional growth corresponding to radiological data was only achieved at a threshold value for periosteal bone formation of 0 MPa, matching observations in animal models. For the following calculations, a Young’s modulus of 10 MPa, *b*/*a* ratio of 0.5, and a threshold for periosteal apposition of 0 MPa are chosen.

**Fig. 5:**
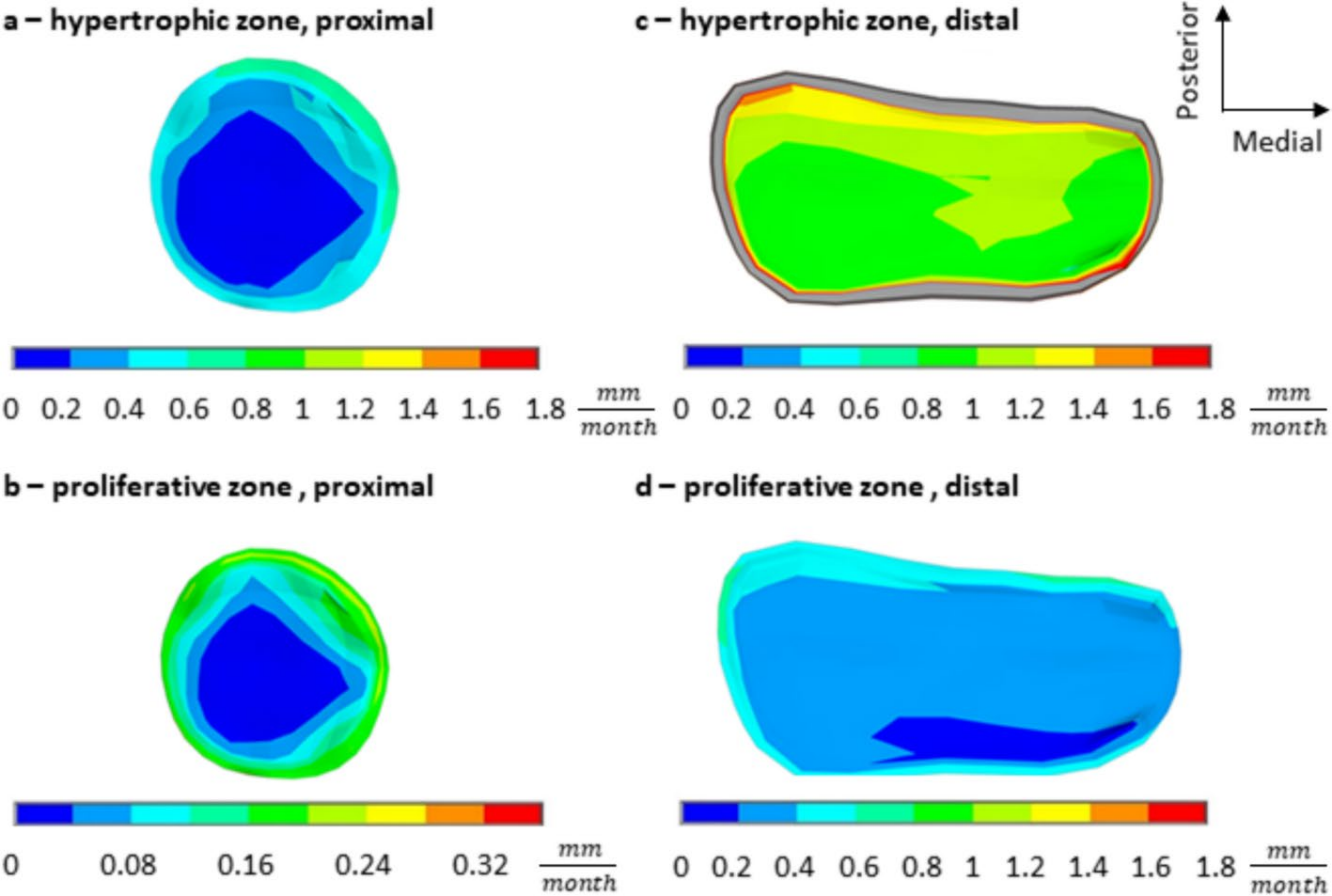
Top view of the proximal hypertrophic (**a**) and proliferative (**b**) zone as well as the distal proximal hypertrophic (**c**) and proliferative (**d**) zone showing the growth rate in mm/month

The growth rate as the sum of the biological baseline and the osteogenic index is calculated for all growth plates (Fig. 5). The growth plate of the proximal femur exhibits a centric distribution of the growth rate. In the growth plate of the distal femur, the growth rate shows a homogeneous distribution.

The simulation predicted an increase in both bone length and diameter in accordance with anthropometric data (Table 2). The ATD remains constant. The NSA decreased on average by 0.1° per month, the anteversion angle by 0.2°. The LDFA decreased by 0.1° per month.

**Table 2:** Changes in the morphometries per year as predicted by the in silico model compared to anthropometric data

Measurement	In silico model	Anthropometric data
Bone length	+ 23.7 mm	+ 20.8 mm [59]
Neck–shaft angle	− 1.4°	−1.1° [60]
Articulo-trochanteric distance	0 mm	0 mm [61]
Lateral distal femoral angle	− 1.6°	0° [62]
Shaft diameter (Anterior–Posterior/Medial–Lateral)	0.7 mm/0.8 mm	1 mm [63]
Anteversion angle	− 2.3°	−0.7° [64]

A second model without modeling the growth of the greater trochanter reduced the decrease of the NSA to 75%. In our model, closure of the apophyseal plate of the greater trochanter results in a higher NSA, matching in vivo data [55, 56].

**Fig. 6:**
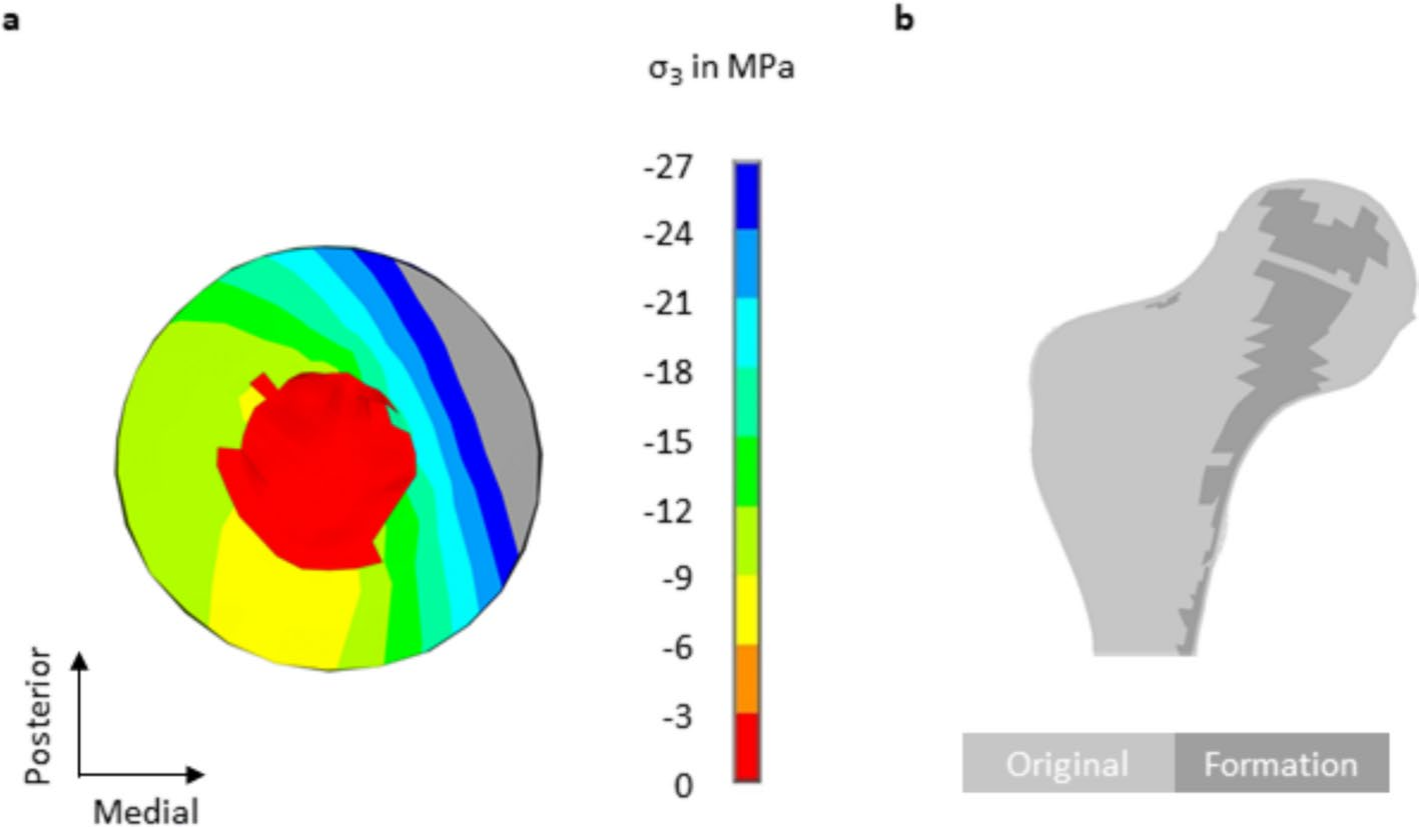
Compressive stresses in transversal section of the femoral shaft (**a**). Increased density due to inner remodeling in the proximal femur. Darker areas correspond to an increased bone mineral density (**b**). Periosteal bone formation is predicted posteriormedially and an increase of cancellous bone density medially

Compressive stresses are highest at the medial side of the femoral shaft (Fig. 6, a). The bone apposition rate is 20, 40, 20, and 60 µm/month anterior, posterior, lateral and medial. This results in a decrease of bending stresses in the sagittal plane of 9% while bending in the frontal plane is essentially unchanged.

Inner remodeling predicted an increase in bone density on the medial side of the proximal femur given by an increase of Young’s modulus (Fig. 6, b). These areas correspond to regions exhibiting higher compression.

## Discussion

Several computational models of femoral long bone growth have been presented in the literature [65–70]. These models provide valuable insights into the principles of the mechanobiology of bone growth. Furthermore, the development of various pediatric deformities can be explained [40–44]. However, they are limited to certain aspects of growth. Complex deformities, such as resulting from fractures, are corrected by a combination of altered growth of all growth plates and bony remodeling [71].

This study investigates stresses acting on the juvenile femur during gait using finite element analysis. A well-established mechanobiological model that uses octahedral shear stresses and hydrostatic stresses as the mechanical input values is employed to calculate the endochondral growth of the proximal and distal femur as well as that of the greater trochanter. Appositional growth of the femoral shaft and inner remodeling of the trabecular bone are also included.

The calculated stresses depend to a large extent on the boundary conditions. The question of bending stress on bones has long been the subject of controversial debate in biomechanics. For adults, Sverdlova and Witzel [72] and later Lutz et al. [73] showed that the femoral shaft is primarily subjected to compressive stresses through muscle activation. This allows weight to be saved, which represents an evolutionary advantage [30]. On the other hand, Bertram and Biewener [74] argue that residual bending allows the direction of bending to be predicted. In this study inertia relief, muscle force optimization, and the iliotibial band are combined to model physiological boundary conditions.

The stress analyses of the juvenile femur reveal reduced bending stresses in the sagittal plane. Nevertheless, bending in the frontal plane cannot be compensated for entirely. The highest appositional growth is predicted for the medial site, but the decrease in the NSA and the growth of the femoral neck result in a more eccentric hip joint resultant. In this simulation the functional adaption compensates the additional bending, but is not able to further reduce bending stresses in the frontal plane. It can be assumed that functional adaption and muscle forces mainly reduce bending in the sagittal plane whereas the iliotibial tract is the primary mechanism for reducing bending stresses in the frontal plane.

However, with a modulus of elasticity of 12,000 MPa for cortical bone, the bones in children and adolescents are significantly more elastic than in adults and their elasticity allows greater bending until material failure. Bones become stiffer as they grow. At the same time, the simulation of bone growth predicts functional changes that reduce bending stresses. The changes in bone morphology during growth can therefore be understood as adaptions to mechanical loading.

In the proximal femur, peak compressive stresses are found medially and result in an increase in bone density similar to previous studies [52]. This shifts the center of mass of the neck of the femur medially toward the resulting axial force. In the diaphysis, internal axial loading is between 200 and 985 N during gait, while shear loading is considerably lower. This is in agreement with comparable studies in adults [75], whereas the weight-adapted axial load is higher. They have important implications in the healing processes of pediatric femoral shaft fractures, as axial loading can enhance fracture healing, while shear loading decelerates fracture healing [76].

The values for changes in bone length, diaphyseal diameter, NSA, and anteversion agree well with anthropometric values. However, the decline in the LDFA is not observed during adolescent growth. A possible explanation might be that the ring of LaCroix, which is not modeled, reduces the deformity of the cartilage in the growth plate. The predicted changes are also a mechanism of functional adaptation, as a decline in the NSA is associated with lower loading on the proximal femur [77]. Growth of the greater trochanter and the femoral neck isthmus could preserve the morphology of the metaphysis during longitudinal growth. The highest increase in cortical thickness is predicted on the medial side of the diaphysis. Indeed, the femoral shaft has a higher thickness on the medial compared to the lateral side [78]. However, the highest cortical bone thickness is observed posterior.

Higher impact loads during running or stair climbing are likely to result in higher posterior compression due to bending, contributing in asymmetric periosteal growth and thereby further building a bending-minimized optimized lightweight structure. Motion capture and ground-reaction forces during different activities in children should be analyzed in further research to add more time-weighted load cases to the model. In conclusion, it could thus be shown that the mechanobiological model of bone growth presented here is suitable for reproducing the physiological growth of the femur with some limitations.

Other limitations of the model include the assumption of constant muscle strength. However, as the angle of the femur changes, the lever arms of the muscles also change, and thereby the vector of the muscle force. Furthermore, the increase in weight leads to an increase in ground reaction force and a higher level of muscle strength due to muscle build-up. Secondarily, this also results in altered hip and knee joint forces. Further developments of the model should therefore include a stronger coupling of the calculations of bony and muscular growth. As discussed above, the simulated load only includes the gait cycle and therefore does not include the maximum loads that occur during sport and play. However, frequent loads are particularly relevant for the morphological adaptation of the bones to the stresses placed on them and gait is subject to less inter-individual variation than sporting activities. These reasons support the choice of gait as a functional load. Also, the cartilaginous growth at the surface of the femoral head and the greater trochanter was not included and periosteal growth was only considered for the diaphysis. Material models are simplified as linear elastic and isotropic and the geometry is simplified. Yadav et al. [69] were able to demonstrate that modeling of patient-specific growth plate geometry increased the accuracy of the predictions. Future research using medical images to develop a patient-specific model and compare the in silico results with the respective radiological follow-up may allow a more detailed validation.

Cortical and cancellous bone has orthotropic material parameters, which are of particular interest for the simulation of bone remodeling and the development of the trabecular columns and Ward’s triangle in the proximal femur. Including orthotropic remodeling rules [79] in the model presented here is promising in further simulating and understanding the endochondral ossification at the growth plate of the proximal femur.

Previous studies found more valid results for patient-specific growth plate geometries. The present study used a single femur anatomy. Modeling growth in different patient-specific models could improve the simulation by superior validation as well as more realistic geometry of the apophyseal growth plate. The present study assumes a constant Young’s modulus across all growth plates. As illustrated in Fig. 6, a low Young’s modulus was associated with results that are more realistic for the LDFA. Therefore, one explanation might be local differences in the material parameters of different growth plates. Another possibility is the tension cording of the superficial pes anserinus, which is hypothesized to play a role in the mechanical loading of the growth plates next to the knee [80]. Additionally, including micromotion at the cortical–cancellous bone interface using a frictional contact formulation might result in more realistic local strains compared to the bounded contact used in the present study.

The model contains a constant biological baseline, which in reality is influenced by hormones and cellular senescence. For clinical decision-making, it is often necessary to consider growth till its completion. Further research should aim to simulate a longer period of growth. Including the effects of sex hormones and cellular senescence on the growth plate might allow for modeling fusion of the growth plates. However, in the current model growth is simulated by updating the location of the nodes. This only allows the simulation of growth over a few months; otherwise, element distortions will occur. Manipulating a volume model and remeshing instead of directly manipulating the mesh might be a feasible solution to this problem [46].

## Conclusions

Bone growth in children does not only lead to an increase in length and thickness but also to complex three-dimensional changes in shape. The implementation of all growth plates and periosteal growth in computational simulations of bone growth can contribute to a better understanding of the interaction between morphological changes and functional loads. Optimizing muscle strength and functional adaptation together reduce bending stresses in growing bone in this in silico model. Future research should aim to simulate bone growth in complex deformities and might be a tool to predict bone remodeling after fractures.

## Materials and methods

**Fig. 7:**
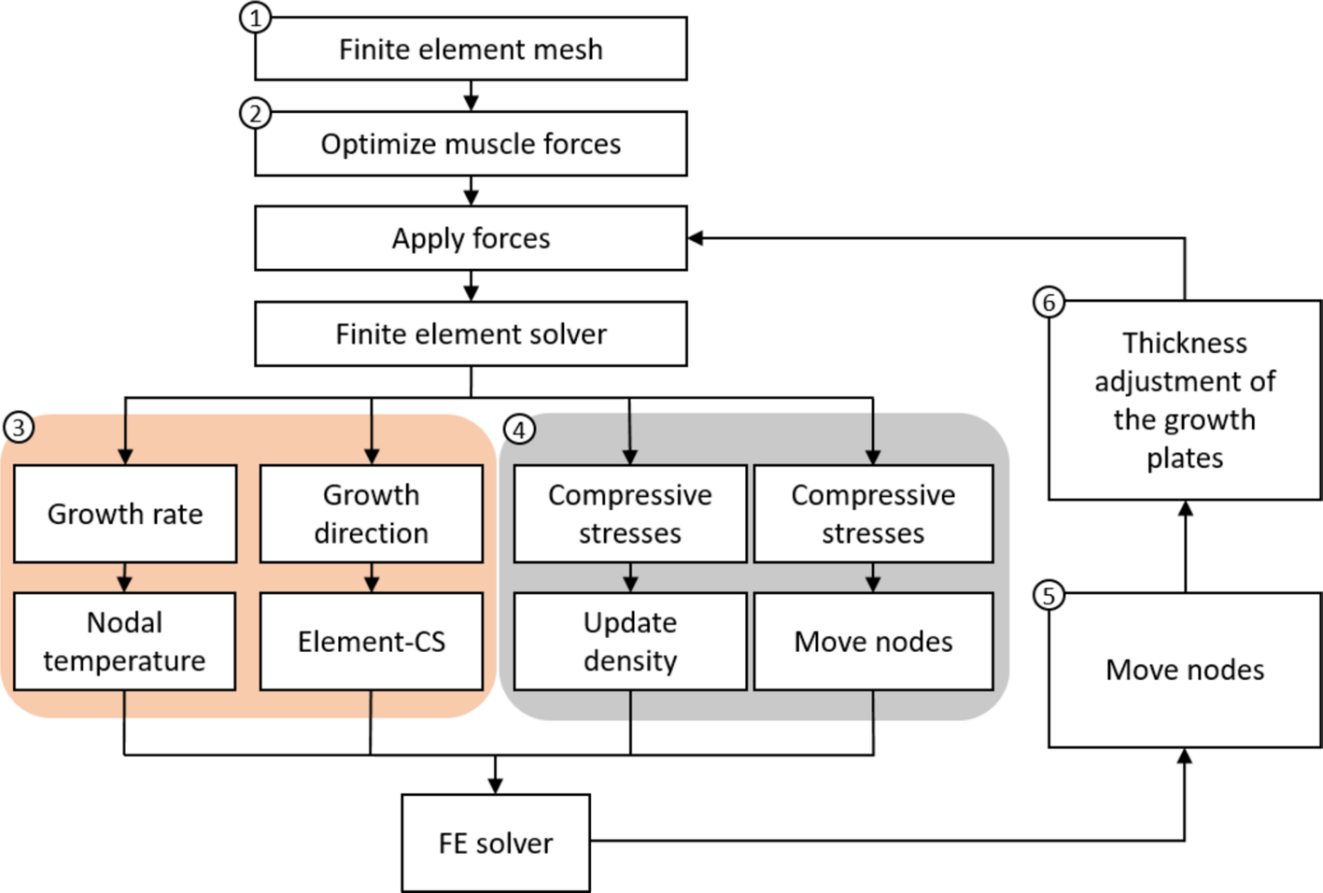
Flow diagram of the mechanobiological model: first, the finite element mesh is established in ANSYS and bending-minimized muscle forces are calculated. Stresses for all load cases are physiologically superimposed and hydrostatic and octahedral shear stresses at the growth plates and 3rd principal stresses in the bone are analyzed. The mechanical stimulus is calculated. The working plane of each growth plate element is aligned with the average deformation during gait. The density of cancellous bone is updated according to Eq. 3 based on 3rd principal stresses. Appositional periosteal growth is modeled by moving the external nodes on a line to the midpoint of the respective transverse plane. Forces are removed and growth rate is applied as temperature, and the model geometry is updated after thermal expansion. Nodes are moved to their final location and the thickness of the growth plates is reset to initial values to model ossification. For the next iteration, forces are applied again to the modified mesh

An iterative algorithm, based on finite element stress analyses, calculation of local growth rates and updating of the model is established (Fig. 7).

1. A finite element mesh of the adolescent right femur, made freely available at SimTK by Kainz et al. [65] based on MRI data of a typically developed child (8 years old, weight: 20.4 kg, height: 1.24 m), was imported into ANSYS Mechanical version 19.2 (ANSYS Inc, Canonsburg, PA, USA). The modeling approach has previously been shown to accurately predict growth in 18 out of 20 cases [81]. The mesh has 22,560 elements and consists of 3-D 8-node structural solids (SOLID185). The element thickness is 4 mm at the shaft and as low as 0.2 mm in regions of interest like the growth plate. A mesh sensitivity analysis is performed. Further mesh refinement up to 902,384 elements results in a change of less than 10% in average principal stresses.

The outer shaft diameter was adapted to 15.5 mm matching values for boys of the age of 8 [63]. The model includes the medullary cavity. The diaphysis is composed of cortical bone, and the meta- and epiphyses are composed of cancellous bone covered by a layer of cortical bone. The proximal and distal growth plates with thicknesses of 0.9 mm and 2.1 mm, respectively, are modeled [82]. The widths are in a ratio of approximately 30:70 to simulate the different contributions of the two epiphyseal plates to femoral growth [83] and correspond to the literature values of an 8-year-old male [84, 85]. Additionally, a layer of elements representing the resting zone is included in the model. The growth plate of the apophysis of the trochanter major is modeled with an angle of 50° to the body horizontal [86]. Pozdnikin et al. [61] reported an almost constant articulo-trochanteric distance measured between the tip of the greater trochanter and the upper point of the femoral head in normal hips of children aged 3 to 17 years. Therefore, the growth plate of the greater trochanter is assumed to have the same thickness as the proximal growth plate to map growth at the same rate. The cartilage of the femoral neck isthmus as well as the articular cartilage is modeled as one layer of proliferative chondrocytes. To reduce computation time and like comparable finite element models [37, 65], all materials are assumed to be linearly elastic, isotropic, and homogeneous. The material properties are given in Table 3. Young´s modulus of the growth plate highly affects the deformation and therefore the growth direction. Values in the literature range, starting with 0.49 MPa for the proliferative and hypertrophic zone and 0.99 MPa for the resting zone [87]. Therefore, a sensitivity analysis is performed to investigate the influence of the rigidity of the growth plates on growth tendency.

**Table 3:** Initial material properties

Material	Density in kg/m^3	Young's modulus in MPa	Poisson's ratio
Cortical bone	1100 [88]	12,000 [90]	0.3 [93]
Cancellous bone	150 [88]	345 [91]	0.25 [9]
Cartilage	1100 [89]	0.5 – 10 [37, 65, 87]	0.47 [87]
Bone marrow	1000 [89]	0.01 [92]	0.49 [92]

Rigid boundary conditions with fixed bearings in all directions at the condyles lead to artificially high stresses [94]. Therefore, physiological boundary conditions with a bearing fixed in all directions at the femoral notch, a bearing fixed in the anterior–posterior direction at the lateral epicondyle of the distal femur, and a bearing fixed in the anterior–posterior and medial–lateral directions at the middle of the femoral head were used [72]. To prevent artificial stresses near the bearings and to take into account the inertia forces generated during movement, inertia relief is used. This method can be used to balance the forces when analyzing individual load steps during the gait and achieve physiological stresses [95]. The boundary conditions are thereby only used to prevent rigid body movements, but the bearings themselves do not generate a reaction force. Instead, the applied forces are balanced by the inertial forces that the body experiences in the acceleration field. The acceleration field is defined as the standard gravitational force on the earth in the inferior direction. To model bone growth by thermal expansion, the femur was fixed at both condyles in all directions.

2. Using load cases, a dynamic gait cycle can be represented by a finite number of static simulations. These discrete load cases can be combined as a functional load history to calculate the mechanical stimulus [26]. For this purpose, a physiological superposition of all loading cases is performed by cumulating the highest values of compressive stresses occurring in one of the loading cases [36]. Nine load cases based on a published multibody simulation are identified and used as initial muscle and joint forces [65]. In addition, the force of the iliotibial band is included, which reduces bending stresses in the diaphysis. This force is taken into account with 50% of the amount of the hip joint resultant of the respective load case [86].

Optimization of muscle forces has been shown to reduce bending stresses, and bending minimization can be used as a principle for determining muscle forces [72]. Therefore, the ANSYS subproblem routine is performed with 30 iterations for each load case. The muscle forces are used as design variables and are allowed to vary within a range of 50 to 200% of the initial value. Forces acting on the hip and knee joint are adopted as fixed values. Bending stresses are calculated in the mediolateral and anteroposterior directions by analyzing the absolute highest principal stresses at four points in the middle of the shaft [96]:

$${\sigma}_{bending}=\pm \left|\frac{{\sigma}_{medial/anterior}-{\sigma}_{lateral/posterior}}{2}\right|.$$

The sum of both bending stresses at the midshaft is minimized (objective function). Additionally, both are independently used as state variables with a target value below 10 MPa. Principal stresses are analyzed by superposition of the minimal and maximal principal stresses, respectively. To calculate internal axial loading, a surface perpendicular to the femoral shaft axis is created. Normal stresses on this surface are averaged and multiplied with the cross-sectional area to obtain axial forces. Values are converted to percent body weight.

3. Epiphyseal growth is modeled as thermal expansion. Proliferation increases the overall volume and therefore accounts for the isotropic growth ratio, while hypertrophy enlarges the columns unidirectionally. A combination of biomechanical and histological analyses found a high correlation between the direction of the distortion of the hypertrophic chondrocytes and orientation of the columns of the growth plate, a mechanism by which shear stresses are reduced [22]. Therefore, for every element representing hypertrophic chondrocytes, the average deformation over all nine load cases is aligned with the z-axis of the element’s local coordinate system. To avoid disturbance of adjacent elements, the vector is calculated for each pair of nodes of the elements of the hypertrophic zone. This is achieved by making use of the fact that in the hexagonal mesh, each proximal node has an assigned distal node. The vector is calculated based on the coordinates of the node adjacent to the proliferative zone and the corresponding node adjacent to the bone as the mean of all nine load cases.

The growth rate is the sum of the biological baseline *r*_*b0*_ and a mechanically stimulated growth rate *r*_*m*_ and is modeled as temperature difference. The growth of an element of the growth plate *g* can be calculated by the local thickness of the respective zone *l* and both growth rates as:

$$g=l\bullet \left({r}_{b0}+{r}_{m}\right).$$

Proliferation and production of extracellular matrix in the proliferative zone account for about 27%, and hypertrophy and production of extracellular matrix in the hypertrophic zone account for 73% [97]. Both growth mechanisms are modeled by the choice of thermal expansion coefficients. r_b0_ and r_m_ are calculated based on a model developed by Wong and Carter [66], Stevens et al. [67], and Yadav et al. [68–70]. Briefly, based on a reported growth rate of the proximal femur of 9 mm/year [80], an assumed percentage of nonmechanically stimulated growth of 66% [16, 68], and a thickness of the proximal growth plate of 0.9 mm, r_b0_ is given as 0.55 mm/month. r_m_ is given by the sum of the maximum octahedral shear stress and the minimum hydrostatic stress obtained over all nine loading cases, where the coefficients a and b describe the respective influence of octahedral shear stress and hydrostatic stress on r_m_ [66]:

$${r}_{m}=a\bullet max\left[\frac{\sqrt{{\left({\sigma}_{1}-{\sigma}_{2}\right)}^{2}+{\left({\sigma}_{2}-{\sigma}_{3}\right)}^{2}+{\left({\sigma}_{3}-{\sigma}_{1}\right)}^{2}}}{3}\right]+b\bullet min\left[\frac{{\sigma}_{1}+{\sigma}_{2}+{\sigma}_{3}}{3}\right].$$

The ratio of b/a was set at 0.5 in agreement with previous studies [40] and calibrated to correspond to 33% of total growth.

4. Changes in bone density in cancellous bone and thereby changes in Young’s modulus can be described by a differential equation developed by Li et al. [98]. It considers bone resorption due to disuse, bone formation due to physiological overload in a dose-dependent manner, and bone resorption due to microdamage and pathological overload. 2 MPa is used as thresholds for bone formation [30]. Pathological overload is assumed for loading four times higher [31, 34]. Furthermore, it is assumed that the curve is symmetrical. This can be used to solve a system of equations for 3 support points. The maximum bone formation rate per bone volume in trabecular bone is 4% per month [99]. The Young’s modulus of a specific element representing cancellous bone after one month of simulated loading E_i+1_ can therefore be expressed as function of the volume at the beginning of the iteration E_i_:

$${E}_{i+1}={E}_{i}+\left(0.2916\bullet {e}^{0.7702 \frac{1}{MPa}{ \sigma }_{3}-0.0772 \frac{1}{{MPa}^{2}}{{ \sigma }_{3}}^{2}}-1\right)\bullet 0.04 MPa.$$

Physiological loading of cortical bone is around ten times higher than in cancellous bone [30]. In contrast to cancellous bone and the endosteal surface, animal models found no periosteal resorption even without any loading [100]. With a maximal apposition rate of 0.06 mm/month [9], the mechanically stimulated apposition rate per month *AR* is given by:

$$AR=\left({e}^{0.0346574 \frac{1}{MPa} {\sigma}_{3}-0.000433217{{\frac{1}{{MPa}^{2}} \sigma }_{3}}^{2}}-1\right)\bullet 0.06 mm/month.$$

A parameter sensitivity study is performed for the Young’s modulus of the growth plate (0.5 to 10 MPa) [37, 64, 84], *b*/*a* ratio (0.3 to 1) [40], and the threshold of periosteal apposition (0 to 20 MPa). 0 MPa is chosen to simulate a remodeling law where no resorption due to unloading takes place as seen in animal models [100], while 20 MPa is the threshold in adults [30, 31].

5. After calculating all growth rates, the nodes are moved to their updated positions according to the growth rates calculated in the previous steps.

6. In a final step, thickness of the growth plate is adjusted to starting values again. This represents the ossification front and ensures the normalization of the growth rate, which is dependent on the thickness of the growth plate.

**Fig. 8:**
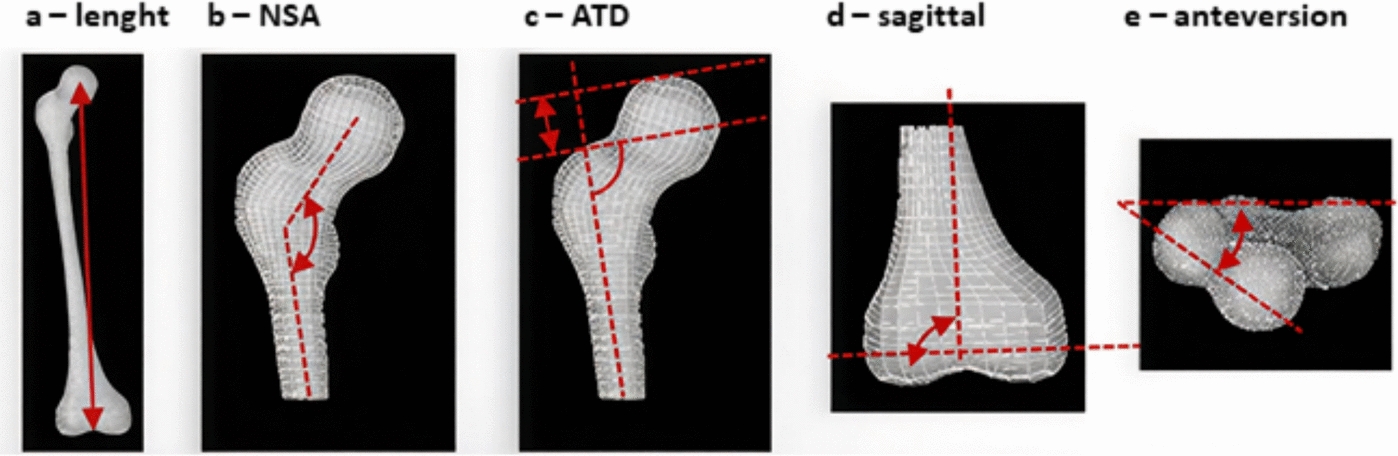
Anthropometric measurements of the femur used in the present study. **a** Length between the center of the femoral head and the femoral notch. **b** Neck–shaft angle (NSA). **c** Articulo-trochanteric distance (ATD). **d** Lateral distal femoral angle (LDFA). **e** Anteversion angle

The updates mesh serves as an initial model for the next iteration. After simulation of bone growth, the following values were measured and compared to reference values for validation (Fig. 8):

•Femur length: the distance between the center of the femoral head and the intercondylar femoral fossa is used here, as these two points are also used to determine the mechanical femoral axis [101].
•Neck–shaft angle (NSA): the projected NSA angle is defined in the frontal plane as the angle between the femoral neck axis and the femoral shaft axis. To determine this, a circle is drawn around the center of the femoral head that intersects the femoral neck medially and laterally. The femoral neck axis can then be constructed by drawing a line through the middle of this line and the center of the head. The femoral shaft axis is the line between the centers of two transverse diameters below the lesser trochanter [102].
•Articulo-trochanteric distance (ATD): the articulo-trochanteric distance is calculated as the distance between two parallel lines orthograde to the femoral shaft axis that are tangential to the maximum points of the femoral head and the greater trochanter [103].
•Lateral distal femoral angle (LDFA): the mechanical lateral distal femoral angle is the lateral angle between the mechanical femoral axis and the knee line as a tangent to the most distal points of both knee condyles. There is also the anatomical lateral distal femoral angle between the femoral shaft axis and the knee line, but this is less commonly used and is therefore not used in this study [104].
•Antetorsion angle (AT): the antetorsion angle describes the rotation of the femoral neck in relation to the shaft. It is measured in the axial plane between the projection of the femoral neck axis and a line tangential to the distal condyles [105].
•Anteroposterior and mediolateral diameter: since the femoral shaft is not cylindrical and shows an eccentric growth behavior, the diameter is determined at the level of the middle of the femur in the axial section between the most anterior and posterior as well as medial and lateral points.

## Data Availability

The data presented in this study are available on request from the corresponding author.
